# Comparison of energy-restricted very low-carbohydrate and low-fat diets on weight loss and body composition in overweight men and women

**DOI:** 10.1186/1743-7075-1-13

**Published:** 2004-11-08

**Authors:** JS Volek, MJ Sharman, AL Gómez, DA Judelson, MR Rubin, G Watson, B Sokmen, R Silvestre, DN French, WJ Kraemer

**Affiliations:** 1 Human Performance Laboratory, Department of Kinesiology, University of Connecticut, 2095 Hillside Road, Unit-1110, Storrs, CT 06269-1110, USA

**Keywords:** weight loss, Atkins diet, hormones, abdominal fat, regional body composition, low-carbohydrate diet

## Abstract

**Objective:**

To compare the effects of isocaloric, energy-restricted very low-carbohydrate ketogenic (VLCK) and low-fat (LF) diets on weight loss, body composition, trunk fat mass, and resting energy expenditure (REE) in overweight/obese men and women.

**Design:**

Randomized, balanced, two diet period clinical intervention study. Subjects were prescribed two energy-restricted (-500 kcal/day) diets: a VLCK diet with a goal to decrease carbohydrate levels below 10% of energy and induce ketosis and a LF diet with a goal similar to national recommendations (%carbohydrate:fat:protein = ~60:25:15%).

**Subjects:**

15 healthy, overweight/obese men (mean ± s.e.m.: age 33.2 ± 2.9 y, body mass 109.1 ± 4.6 kg, body mass index 34.1 ± 1.1 kg/m^2^) and 13 premenopausal women (age 34.0 ± 2.4 y, body mass 76.3 ± 3.6 kg, body mass index 29.6 ± 1.1 kg/m^2^).

**Measurements:**

Weight loss, body composition, trunk fat (by dual-energy X-ray absorptiometry), and resting energy expenditure (REE) were determined at baseline and after each diet intervention. Data were analyzed for between group differences considering the first diet phase only and within group differences considering the response to both diets within each person.

**Results:**

Actual nutrient intakes from food records during the VLCK (%carbohydrate:fat:protein = ~9:63:28%) and the LF (~58:22:20%) were significantly different. Dietary energy was restricted, but was slightly higher during the VLCK (1855 kcal/day) compared to the LF (1562 kcal/day) diet for men. Both between and within group comparisons revealed a distinct advantage of a VLCK over a LF diet for weight loss, total fat loss, and trunk fat loss for men (despite significantly greater energy intake). The majority of women also responded more favorably to the VLCK diet, especially in terms of trunk fat loss. The greater reduction in trunk fat was not merely due to the greater total fat loss, because the ratio of trunk fat/total fat was also significantly reduced during the VLCK diet in men and women. Absolute REE (kcal/day) was decreased with both diets as expected, but REE expressed relative to body mass (kcal/kg), was better maintained on the VLCK diet for men only. Individual responses clearly show the majority of men and women experience greater weight and fat loss on a VLCK than a LF diet.

**Conclusion:**

This study shows a clear benefit of a VLCK over LF diet for short-term body weight and fat loss, especially in men. A preferential loss of fat in the trunk region with a VLCK diet is novel and potentially clinically significant but requires further validation. These data provide additional support for the concept of metabolic advantage with diets representing extremes in macronutrient distribution.

## Introduction

Recent reports showing a greater weight loss with a free-living very low-carbohydrate ketogenic (VLCK) than a low-fat diet after 3 and 6 months [1-5] has generated interest in mechanisms that may account for these responses. Earlier work that involved comparison of isocaloric formula VLCK and low-fat (LF) diets [6], indicated that weight loss was greater with a VLCK, suggesting a metabolic advantage (i.e., a greater weight loss with one diet over another with different macronutrient distribution but the same energy content) [7,8].

Although several studies have shown that VLCK diets result in greater reductions in body mass, it remains unclear how these diets affect the composition of weight loss and the distribution of fat loss. Some early reports show that VLCK diets result in preferential loss of fat and preservation of lean body mass [9-12], suggestive of a nutrient partitioning effect. In accordance with this notion, we recently reported that a free-living 6-week VLCK diet prescribed to be isoenergetic resulted in significant decreases in fat mass and increases in lean body mass in normal-weight men [13]. However, other studies have not shown a preferential loss of fat on a VLCK diet [14]. No studies have examined the effects of a VLCK diet on the distribution of fat loss. Since accumulation of fat in the abdominal area is associated with insulin resistance, diabetes, dyslipidemias and atherosclerosis [15], demonstration of the effects of a VLCK diet on regional fat distribution is important.

Volek and Westman [16] have reviewed the potential favorable effects of VLCK diets while other reviews that have focused on the potential adverse effects of VLCK diets caution to avoid or limit their use [17-19]. Given the varying opinions in respect to VLCK diets, we thought it was important to provide additional information related to the effects of a VLCK diet on weight loss, body composition, and regional fat distribution. We previously reported that a VLCK diet has favorable effects on biomarkers for cardiovascular disease [20-22]. The primary purpose of this investigation was to compare the effects of isocaloric, energy-restricted (-500 kcal/day from estimated needs to maintain weight) VLCK and LF diets on weight loss, body composition, trunk fat, and REE in overweight men and women.

## Methods

### Subjects

A total of twenty-eight healthy volunteers (15 men and 13 women) were recruited by flyers and word-of-mouth. Subjects were between 20 and 55 y, nonsmokers, and greater than 25 percent body fat determined via dual-energy X-ray absorptiometry (DEXA). Subjects went through a thorough screening procedure to ensure they would be committed to completing the study. Exclusion criteria included a body mass >145 kg (because of technical difficulties in performing DEXA), post-menopausal women, overt diabetes, cardiovascular, respiratory, gastrointestinal, thyroid or any other metabolic disease, weight change ± 2 kg over the last month, adherence to special diets, use of nutritional supplements (except a daily multi-vitamin/mineral), and use of medications to control blood lipids or glucose. The majority of subjects were sedentary and were instructed not to start an exercise program during the study. Those who were active were instructed to maintain the same level of physical activity throughout the study. Baseline characteristics of men and women stratified by diet order are shown in Table 1 (see additional file 1). The study was conducted in accordance with the guidelines of the Institutional Review Board at the University of Connecticut.

### Experimental Approach

Our primary research question was to compare VLCK and LF diets on weight loss, fat loss, and trunk fat loss. We addressed this in several ways. First, subjects were initially randomly assigned to either a LF or VLCK weight loss diet. Weight loss, body composition (fat mass and lean body mass), trunk fat, and resting energy expenditure (REE) were assessed before and after each diet (Phase I). Because there is often a great deal of variation in response to diet, we decided that a direct comparison of responses to a VLCK and LF diet should be made in the same person. To achieve this aim, we asked subjects to switch to the opposite diet after completion of the first diet period (Phase II), after which the same measurements were assessed (i.e., each subject consumed a VLCK and LF diet). This experimental approach allowed us to compare these two diets in two ways: a *between *group comparison of subjects who either consumed a VLCK or LF diet during Phase I, and a *within *group comparison of subjects who consumed both a VLCK and LF diet. The within group comparison was further analyzed to determine if the order of diets had any effect on the responses. Subjects kept detailed food diaries during three 1 wk periods (21 days total) of each diet. Men consumed each diet for 50 days whereas women consumed the diets for approximately 30 days in order to control for possible effects of menstrual phase on some of the dependent variables measured in this study [23,24]. All testing for women was performed between days 2–4 of the follicular phase as self-reported by the women.

### Diet Interventions

Both experimental diets were designed to be hypoenergetic (-500 kcal/day). Energy levels were assigned to the nearest 200 kcal increment based on REE obtained using indirect calorimetry at the start of the study and appropriate activity factors. Standard diabetic exchange lists were used to ensure a constant energy and macronutrient balance of protein (~20% energy), fat (~25% energy), and carbohydrate (~55% of energy) during the LF diet. The LF diet was also designed to contain <10% saturated fat and <300 mg cholesterol (i.e., a Step-I diet). Foods encouraged during the LF diet included whole grains (breads, cereals, and pastas), fruit/fruit juices, vegetables, vegetable oils, and low-fat dairy and meat products. We developed customized diabetic exchange lists for the VLCK diet period in order to ensure a constant energy and balance of protein (~30% energy), fat (~60% energy), and carbohydrate (~10% of energy) throughout the day. There were no restrictions on the type of fat from saturated and unsaturated sources or cholesterol levels. Foods commonly consumed on the VLCK diet were beef (e.g., hamburger, steak), poultry (e.g., chicken, turkey), fish, oils, various nuts/seeds and peanut butter, moderate amounts of vegetables, salads with low-carbohydrate dressing, moderate amounts of cheese, eggs, protein powder, and water or low-carbohydrate diet drinks. Low-carbohydrate bars and shakes (Atkins Nutritionals, Inc., Hauppauge, NY) were provided to subjects during the VLC diet. A daily multi-vitamin/mineral complex that provided micronutrients at levels ≤ 100% of the RDA was given to subjects during both experimental diets.

All subjects received extensive initial instruction and follow-up by registered dietitians on how to translate foods/meals into diabetic exchanges. Subjects were also provided with a packet outlining specific lists of appropriate foods, recipes, and sample meal plans that were compatible with their individual preferences for both experimental diets. Subjects received thorough instructions for completing detailed weighed food records during three 7-day periods (21 days total) for each diet. Food measuring utensils and scales were provided to subjects to ensure accurate reporting of food/beverage amounts consumed. Food diaries were analyzed for energy and macro/micronutrient content (NUTRITIONIST PRO™, Version 1.3, First Databank Inc, The Hearst Corporation, San Bruno, CA). The program had no missing values for the nutrients reported. The database was extensively modified by our group to include new foods and recipes.

To ensure that carbohydrates were restricted throughout the VLCK diet, subjects tested their urine daily using reagent strips (Bayer Corporation, Elkhart, IN) at the same time of day and recorded the result on log sheets. The test is specific for acetoacetic acid, which produces a relative color change when it reacts with nitroprusside. We have found this to be a very sensitive indicator of carbohydrate restriction and compliance to a VLCK diet in our prior studies [13,21,22,25]. Subjects were required to report to the laboratory each week to monitor weight, dietary compliance, and check the level of ketones (during the VLCK diet only). Subjects received follow-up counseling and dietetic education in necessary.

### Body Mass and Body Composition

Body mass and body composition were measured in the morning after a 12 h overnight fast. Body mass was recorded to the nearest 100 g on a digital scale (OHAUS Corp., Florham Park, NJ) with subjects either nude or wearing only underwear. Whole body and regional body composition were assessed using a fan-beam DEXA (Prodigy™, Lunar Corporation, Madison, WI). Regional analysis of the trunk was assessed according to anatomical landmarks by the same technician using computer algorithms (enCORE version 6.00.270). Coefficients of variation for lean body mass, fat mass, and bone mineral content on repeat scans with repositioning on a group of men and women in our laboratory were 0.4, 1.4, and 0.6%, respectively.

### Resting Energy Expenditure

Resting energy expenditure measurements were made by indirect calorimetry (MedGraphics CPX/D, Medical Graphics Corporation, St. Paul, MN) after an overnight fast (>12 h) with subjects resting supine in comfortable thermoneutral conditions. The metabolic cart was calibrated with a standard gas mixture each morning. Subjects were instructed to relax quietly in a dimly lit room without sleeping for 30 min and oxygen consumption (VO_2_) and VCO_2 _were averaged during the last 20 min for determination of REE [26]. We assessed reliability on two subjects who were tested two times per day for six consecutive days. The coefficient of variation for REE (kJ/day) was 2.95% for duplicate measures on the same day and 6.20% between days.

### Statistical Analysis

Changes in body weight, body composition, and REE between diets were assessed using independent t-tests for between group comparisons (i.e., Phase I responses) and dependent t-tests were used to assess within group comparisons. All statistical analyses were performed with Statistica 5.5 for windows (StatSoft Inc, Tulsa, OK). Significance was set at *P *≤ 0.05.

## Results

### Dietary nutrient intakes (Table 2)

There were no differences in dietary nutrient intakes between groups at baseline. Subjects complied very well with the given instructions for both diet interventions according to analysis of diets records. During the diet interventions, all dietary nutrients were significantly different between the VLCK and LF diets with the exception of total dietary energy (women only) and alcohol (see additional file 1 Table 2). Dietary energy was higher during the VLCK than the LF diet in men. We achieved our goals for each diet with <25% of total energy coming from fat on the LF diet and <10% of total energy coming from carbohydrate on the VLCK diet. All subjects were in ketosis throughout the VLCK diet as indicated by color changes on the urinary reagent strips (data not shown), indicating compliance in terms of carbohydrate restriction.

### Between group comparison of subjects who either consumed a VLCK or LF diet

**Figure 1:**
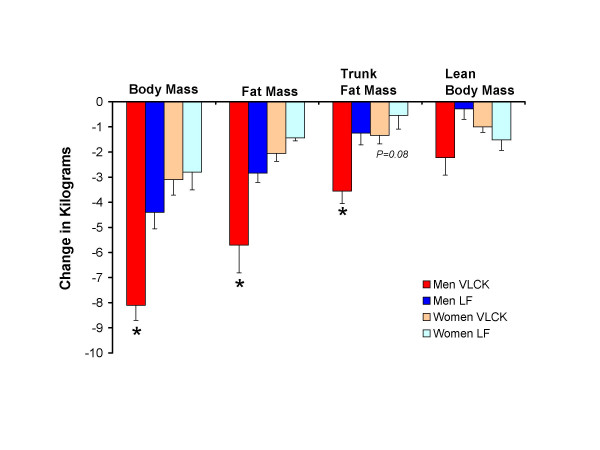
Mean decreases in body mass, total fat mass, trunk fat mass, and lean body mass in men who consumed a very low-carbohydrate ketogenic (VLCK) diet (*n *= 8) or a low-fat (LF) diet and in women who consumed a VLCK (*n *= 7) and LF (*n *= 6) diet. *P < 0.05 from LF change in men (independent t-test).

The reductions in body mass, total fat mass, and trunk fat mass were significantly greater after the VLCK than the LF diet for men, but not for women (Fig 1). The greater reduction in trunk fat was not merely due to the greater total fat loss in men, because the ratio of trunk fat/total fat was also significantly reduced during the VLCK diet in men (VLCK 57.9 ± 1.8 to 57.1 ± 1.7%; LF 60.2 ± 1.3 to 61.4 ± 1.1%). Although the ratio of trunk fat/total fat in women was reduced more on the VLCK diet (51.9 ± 2.4 to 51.2 ± 2.3%) compared to the LF diet (44.2 ± 2.2 to 44.5 ± 2.3%), this was not significant. There were no significant differences in REE expressed in absolute terms between the VLCK diet (men 2005 ± 283 to 1865 ± 96; women 1177 ± 43 to 1161 ± 101 kcal/day) and the LF diet (men 2352 ± 316 to 2119 to 137; women 1319 ± 92 to 1224 ± 100 kcal/day). Expressed relative to body mass, REE was maintained in men consuming the VLCK diet (19.6 ± 0.7 to 19.8 ± 0.7 kcal/kg) but decreased on the LF diet (20.4 ± 1.0 to 19.0 ± 0.8 kcal/kg). As expected, the respiratory exchange ratio decreased on the VLCK compared to the LF diet further indicating compliance to the VLCK diet.

### Within group comparison of subjects who consumed both a VLCK and LF diet

**Figure 2:**
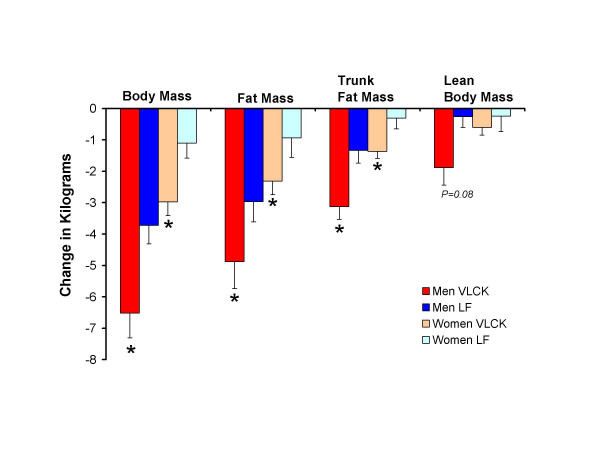
Mean decreases in body mass, total fat mass, trunk fat mass, and lean body mass in men (*n *= 15) and women (*n *= 13) who consumed both a very low-carbohydrate ketogenic (VLCK) and a low-fat (LF) diet in a randomized and balanced fashion. *P < 0.05 from LF change (dependent t-test).

**Figure 3:**
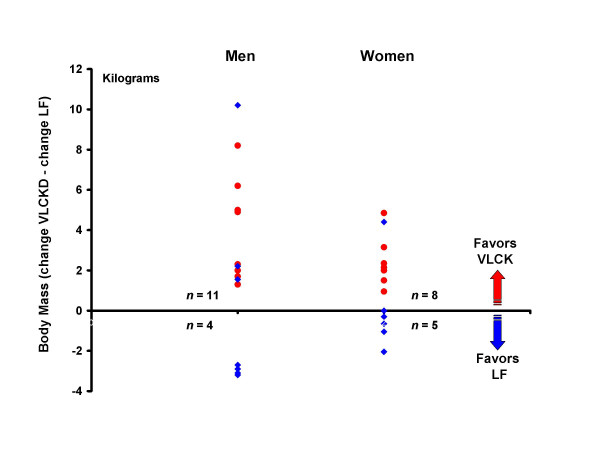
Individual differences between weight loss on a very low-carbohydrate ketogenic (VLCK) diet minus weight loss on a low-fat (LF) diet for each person. Positive numbers reflect greater weight loss on the VLCK, whereas negative numbers indicate greater weight loss on the LF diet. Red circles = order of diets VLCK then LF. Blue diamonds = order of diets LF then VLCK.

**Figure 4:**
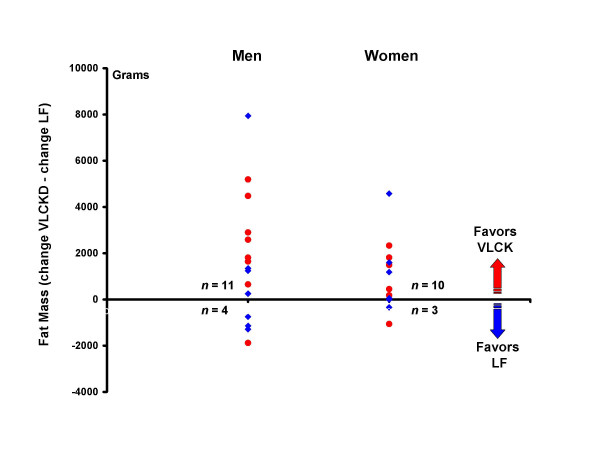
Individual differences between total fat loss on a very low-carbohydrate ketogenic (VLCK) diet minus total fat loss on a low-fat (LF) diet for each person. Positive numbers reflect greater weight loss on the VLCK, whereas negative numbers indicate greater weight loss on the LF diet. Red circles = order of diets VLCK then LF. Blue diamonds = order of diets LF then VLCK.

**Figure 5:**
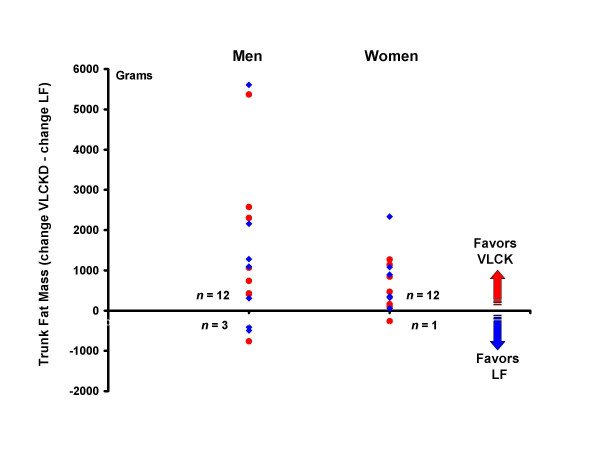
Individual differences between trunk fat loss on a very low-carbohydrate ketogenic (VLCK) diet minus trunk fat loss on a low-fat (LF) diet for each person. Positive numbers reflect greater weight loss on the VLCK, whereas negative numbers indicate greater weight loss on the LF diet. Red circles = order of diets VLCK then LF. Blue diamonds = order of diets LF then VLCK.

Dependent t-tests were used to assess the difference between changes on the VLCK and LF diets. Again, the VLCK diet resulted in significantly greater reductions in body mass, total fat mass, and trunk fat mass for men. For these variables, the reductions were also significantly greater in women, in contrast to the results from between group comparisons (Fig 2). Individual data showing the comparison between diets for each person is shown for body mass (Fig 3), total fat mass (Fig 4), and trunk fat mass (Fig 5). In men, a majority benefited more from the VLCKD in terms of weight loss (11/15 subjects), total fat loss (11/15 subjects), and trunk fat loss (12/15 subjects). In women, a majority also benefited more from the VLCK diet in terms of weight loss (8/13 subjects), total fat loss (10/13 subjects), and trunk fat loss (12/13 subjects). It is noteworthy that 5 men showed more than a 10 pound difference in weight loss when the diets were compared. There was a preferential loss of fat in the trunk region as evidenced by significantly greater reduction in the ratio of trunk fat to total body fat after the VLCKD in both men and women. There were no significant differences in REE responses between diets.

**Figure 6:**
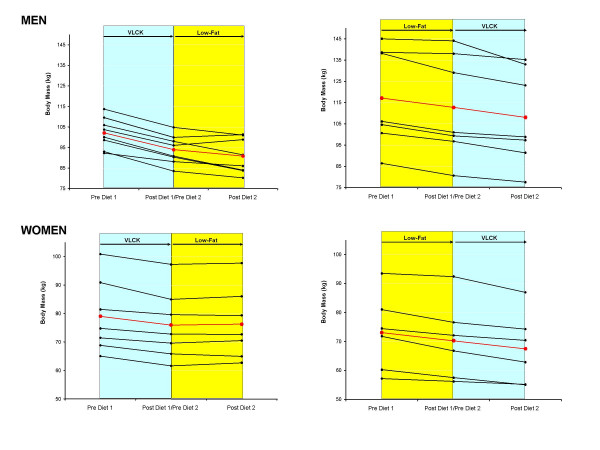
Individual changes in body mass in men (upper panels) and women (lower panels) who started on a very low-carbohydrate ketogenic (VLCK) and switched to a low-fat (LF) diet (left panels) and vice versa (right panels). Mean response is shown in red.

**Figure 7:**
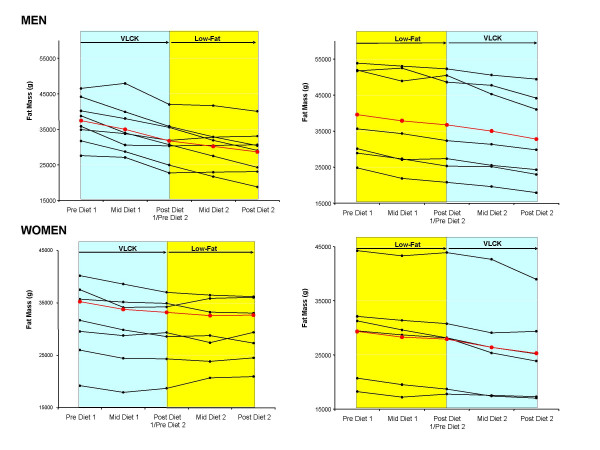
Individual changes in total fat mass in men (upper panels) and women (lower panels) who started on a very low-carbohydrate ketogenic (VLCK) and switched to a low-fat (LF) diet (left panels) and vice versa (right panels). Mean response is shown in red.

**Figure 8:**
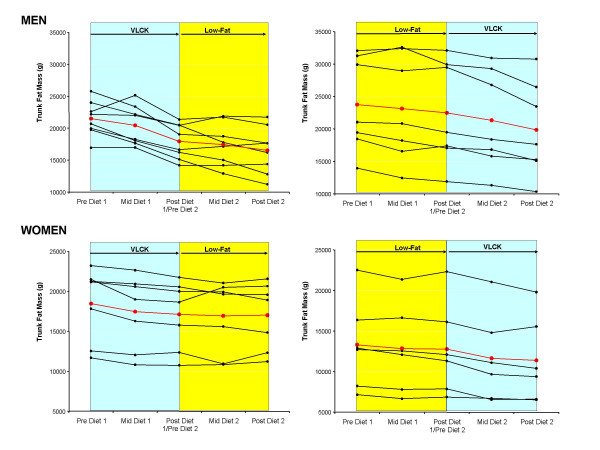
Individual changes in trunk fat mass in men (upper panels) and women (lower panels) who started on a very low-carbohydrate ketogenic (VLCK) and switched to a low-fat (LF) diet (left panels) and vice versa (right panels). Mean response is shown in red.

The results presented thus far indicate that VLCK diets result in superior weight loss and fat loss in men, and to a lesser extent in women, compared to a low-fat diet. To determine if this finding was influenced by the order the diets were implemented, we compared the responses to both diets between those who consumed the VLCK diet first to those who consumed the LF diet first. The individual responses to both diets over time are shown for body mass (Fig 6), total fat mass (Fig 7), and trunk fat mass (Fig 8). Statistically comparing the responses to a VLCK and LF diet within subjects, the only variable that was significantly affected by the order of the diet was body mass. In other words, the advantage of the VLCK over the LF diet was more dramatic for those who started the VLCK first. The individual responses reveal that three men and four women who did VLCK first, actually regained body mass and fat mass after the switch to the LF diet, whereas no subjects regained weight or fat mass after switching to the VLCK diet.

## Discussion

We previously reported superior responses with a VLCK over a LF diet in a number of cardiovascular risk factors in these subjects [25,27]. The results of this study demonstrate that short-term VLCK diets also outperform LF diets in terms of weight loss and fat loss. These effects occurred despite apparently similar energy deficits between diets and in the case of men, significantly greater energy intake. Greater weight loss with a VLCK over a LF diet is consistent with the findings from other studies, and provides further support for the concept of metabolic advantage [7,8]. Since food was not provided this conclusion cannot be made with certainty, but we find it highly unlikely that any potential error in quantifying energy intake would account for the dramatic differences in weight and fat loss between diets. We can say with confidence that we studied subjects that were restricting carbohydrates to very low levels as verified by dietary food records, urine ketones, and low resting respiratory exchange ratios obtained with indirect calorimetry.

The basic principle on which weight loss diets are based is to reduce dietary energy intake below energy expenditure. Whether the relative composition of macronutrients can influence the magnitude or composition of weight loss achieved on an energy-restricted diet has been a point of contention. Several comparisons of isocaloric VLCK and LF diets, like the current report, show greater weight loss on a VLCK diet [6,16] supporting the long held notion of a metabolic advantage [28]. Given such evidence, it is difficult to understand the alternate position claiming a calorie must be a calorie in order to satisfy the first law of thermodynamics [29]. Although the origin of the difference in weight loss between VLCK and LF diets remains controversial, such a response clearly does not violate any thermodynamic laws [7]. Not all studies have shown greater weight loss with a VLCK diet [30] and the specific conditions that are required to elicit a metabolic advantage remain unknown.

One argument is that the greater weight loss on *ad libitum *VLCK diets is a result of spontaneously reducing energy intake [31], and this has been reported previously [32]. A reduction in energy intake on a VLCK diet has a logical physiologic basis and could account for a portion of the greater weight observed in studies that involved free-living *ab libitum *VLCK diets. Ketone levels increase several-fold on a VLCK diet, and β-hydroxybutyrate (the major circulating ketone body) has been shown to directly inhibit appetite [33]. Also, the low glycemic nature of a VLCKD may prevent transient dips in blood glucose, which can occur with higher carbohydrate diets. Thus, avoidance of hypoglycemic episodes may reduce appetite [34]. In this study we did not report a significantly lower energy intake on the VLCK compared to the LF diet. In fact, a higher energy intake was observed on the VLCK diet in men. In this case, it is often claimed that inaccurate reporting of dietary intake or errors in nutrient databases (e.g., overestimation of calories from certain cuts of meats) account for the greater weight reducing effects of VLCK diets. On the other hand, LF diets are frequently encouraged because of their high bulk and over-reporting seems as likely on a LF as a VLCK diet. In the absence of a clear reason why error in these studies should always go in one direction – LF rarely do better than VLCK – one has to take the data at face value. Also, the large difference in weight loss between men on the VLCK and LF diets in the present study suggests that at least some impact of macronutrient composition is being seen.

Metabolic advantage may occur on a VLCK diet due to the demand on protein turnover for gluconeogenesis [35], greater thermogenic effect of protein and loss of energy as heat [36,37], and/or excretion of energy in the form of ketones via urine, feces, and/or sweat. Although we did not see a difference in REE, the metabolic advantage on a VLCK diet may be below the sensitivity of our measurements. Further, since REE was obtained in a postabsorptive state, this does not rule out a potential benefit derived from the acute postprandial thermic effect of protein ingestion. In terms of REE, there was a slight advantage for men on the VLCK diet when expressed relative to body mass, which could benefit long-term weight maintenance but this needs to be validated in studies of longer duration.

Although the issue of whether VLCK diets result in greater weight loss compared to LF diets has obvious significance, a primary purpose of this study and an equally important question relates to the composition of weight loss. In a meta-analysis, Garrow and Summerbell [38] predict from regression analysis that for a weight loss of 10 kg by dieting alone, the expected loss from fat mass is 71%. The few studies that have assessed body composition suggest that VLCK diets may result in preferential loss of fat mass. Benoit et al. [10] showed that a 10 day VLCK diet (4.2 MJ/day) resulted in a weight loss of -6.6 kg in obese men, 97% of which was fat mass. Young et al. [9] compared the effects of three isoenergetic (7.5 MJ/day), isoprotein (115 g/day) diets containing varying carbohydrate contents (30, 60, and 104 g/day) on weight loss and body composition in obese men. After 9 weeks, weight loss was 16.2, 12.8, and 11.9 kg and fat accounted for 95%, 84%, and 75% of the weight lost, respectively. Willi et al. [11] showed that an 8 week VLCK diet (2.7–3.0 MJ/day) resulted in a weight loss of -15.4 kg and an increase in lean body mass of +1.4 kg in obese adolescents. An 8-week VLCK diet in overweight women resulted in a decrease in body mass of -5 kg, 80% of which was fat mass [12]. Our laboratory recently reported that a 6 week VLCK diet resulted in significant decrease in body mass (-2.2 kg), entirely accounted for by a decrease in fat mass (-3.3 kg) and concomitant increases in lean body mass (+1.1 kg) in normal-weight men [13]. The body composition results from the present study are in closer agreement with predictions from the meta-analysis [38].

A novel and potentially clinically significant finding was a preferential loss of fat in the trunk region with a VLCK diet, which was approximately three-fold greater during the VLCK than the LF diet. Upper body fat carries a greater health risk than fat stored in other regions of the body and thus an effective weight loss approach should consider the regional distribution of fat loss. Proportionally, trunk fat mass comprised less of the total fat mass after the VLCK but not the LF diet. The mechanisms regulating composition of weight loss and distribution of fat loss during VLCK diets remain unclear, but could be mediated in part by changes in hormones such as insulin, leptin, or cortisol that could differentially impact nutrient partitioning.

In summary, this study showed greater weight loss and fat loss preferentially from the trunk region in subjects on a closely monitored free-living VLCK diet compared to a LF diet. These diets were prescribed to be energy restricted and isocaloric. The superiority of the VLCK diet over the LF diet was most dramatic for men, but when individual responses were examined, a group of women clearly showed metabolic advantage as well. Indeed, 12/13 women experienced greater fat loss in the trunk region during the VLCK diet compared to the low-fat diet. Such a response is consistent with a metabolic advantage of VLCK diets. The ultimate proof for such a theory will depend on the findings from carefully controlled feeding and metabolic studies that encompass physiological measurements to isolate plausible mechanisms.

## Supplementary Material

Additional File 1 Table 1. Baseline characteristics of men and women based on their starting diet. Table 2. Daily intakes of dietary energy and nutrients at baseline and during both diets. Click here for file
