# Supplementary material for: Comparison of energy-restricted very low-carbohydrate and low-fat diets on weight loss and body composition in overweight men and women
Source: Nutr Metab (Lond). 2004 Nov 8;1:13. doi: 10.1186/1743-7075-1-13 (PMC538279; doi:10.1186/1743-7075-1-13)
Supplement: Additional File 1 — Table 1. Baseline characteristics of men and women based on their starting diet. Table 2. Daily intakes of dietary energy and nutrients at baseline and during both diets. [file 1743-7075-1-13-S1.doc]

**TABLE 1.** Baseline characteristics of men and women.

**Men Women**

**VLCK→LF LF→VLCK VLCK→LF LF→VLCK**

**(*n* = 8) (*n* = 7) (*n* = 7) (*n* = 6)**

**Characteristic**

Age (y) 34.9 ± 5.2 36.0 ± 2.0 30.5 ± 4.5 28.6 ± 3.4

Height (m) 1.78 ± 0.06 1.80 ± 0.10 1.61 ± 0.06 1.59 ± 0.07

Weight (kg) 102.1 ± 7.6 117.1 ± 23.0 79.0 ± 12.9 73.0 ± 13.4

BMI (kg/m^2^) 32.4 ± 2.3 36.0 ± 5.2 30.5 ± 4.5 28.6 ± 3.4

Body Fat (%)# 36.5 ± 5.3 33.4 ± 5.14 44.3 ± 3.4 39.3 ± 5.7

Trunk Fat/Whole Body Fat (%)# 57.9 ± 5.1 60.2 ± 3.5 51.9 ± 6.4 44.2 ± 5.4*

Values are mean ± SD

VLCK→LF = Very low-carbohydrate followed by low-fat diet. LF→VLCK = Low-fat followed by very low-carbohydrate diet.

#Obtained using dual-energy X-ray absorptiometry.

**P* ≤ 0.05 from women who started VLCK→LF.

**TABLE 2.** Daily intake of dietary energy and nutrients at baseline and during the very low-carbohydrate ketogenic (VLCK) and low-fat (LF) diets.

**Men (n = 15) Women (n = 13)**

**Baseline VLCK LF Baseline VLCK LF**

**Nutrient**

Energy (MJ) 2593 ± 568 1855 ± 432 1562 ± 285* 1931 ± 306 1288 ± 281 1243 ± 291

Protein (g) 106 ± 24 130 ± 30 79 ± 18* 76 ± 16 88 ± 19 59 ± 14*

Protein (%) 16 ± 2 28 ± 5 20 ± 4* 15 ± 2 28 ± 2 19 ± 4*

Carbohydrate (g) 308 ± 77 36 ± 18 224 ± 56* 243 ± 35 29 ± 8 186 ± 44*

Carbohydrate (%) 47 ± 5 8 ± 3 56 ± 7* 50 ± 7 9 ± 2 59 ± 4*

Total Fat (g) 104 ± 28 130 ± 34 39 ± 11* 71 ± 19 88 ± 21 29 ± 8*

Total Fat (%) 35 ± 4 63 ± 4 23 ± 7* 32 ± 4 63 ± 3 21 ± 3*

Saturated Fat (g) 35 ± 12 46 ± 13 13 ± 3* 24 ± 5 34 ± 11 10 ± 3*

Monounsaturated Fat (g) 29 ± 8 48 ± 18 10 ± 5* 16 ± 6 29 ± 10 8 ± 3*

Polyunsaturated Fat (g) 16 ± 4 20 ± 6 6 ± 3* 10 ± 6 12 ± 3 5 ± 1*

Alcohol (%) 2 ± 3 1 ± 2 1 ± 1 2 ± 3 1 ± 1 2 ± 3

Cholesterol (mg) 303 ± 94 731 ± 290 170 ± 66* 272 ± 111 470 ± 192 124 ± 42*

Dietary Fiber (g) 16 ± 5 8 ± 6 17 ± 6* 14 ± 4 8 ± 2 16 ± 4*

Values are mean ± SD

Analysis performed on 7 days of diet records during baseline (habitual diet) and 21 days during the VLCK and LF diets.

**P* ≤ 0.05 between VLCK and LF diets.
